# Response of a neuronal network computational model to infrared neural stimulation

**DOI:** 10.3389/fncom.2022.933818

**Published:** 2022-08-15

**Authors:** Jinzhao Wei, Licong Li, Hao Song, Zhaoning Du, Jianli Yang, Mingsha Zhang, Xiuling Liu

**Affiliations:** ^1^Key Laboratory of Digital Medical Engineering of Hebei, Hebei University, Baoding, China; ^2^College of Electronic and Information Engineering, Hebei University, Baoding, China; ^3^State Key Laboratory of Cognitive Neuroscience and Learning, Beijing Normal University, Beijing, China; ^4^IDG/McGovern Institute for Brain Research at BNU, Beijing Normal University, Beijing, China; ^5^Division of Psychology, Beijing Normal University, Beijing, China

**Keywords:** computational model, infrared neural stimulation, neuronal network, photothermal effect, ionic channel, membrane capacitance

## Abstract

Infrared neural stimulation (INS), as a novel form of neuromodulation, allows modulating the activity of nerve cells through thermally induced capacitive currents and thermal sensitivity ion channels. However, fundamental questions remain about the exact mechanism of INS and how the photothermal effect influences the neural response. Computational neural modeling can provide a powerful methodology for understanding the law of action of INS. We developed a temperature-dependent model of ion channels and membrane capacitance based on the photothermal effect to quantify the effect of INS on the direct response of individual neurons and neuronal networks. The neurons were connected through excitatory and inhibitory synapses and constituted a complex neuronal network model. Our results showed that a slight increase in temperature promoted the neuronal spikes and enhanced network activity, whereas the ultra-temperature inhibited neuronal activity. This biophysically based simulation illustrated the optical dose-dependent biphasic cell response with capacitive current as the core change condition. The computational model provided a new sight to elucidate mechanisms and inform parameter selection of INS.

## Introduction

Many forms of external physical stimulation (electric, optical, ultrasound, and magnetic stimulation) can regulate brain functions and the treatment of brain disorders (Darmani et al., 2022). Compared with electrical stimulation, known as the gold standard (Barborica et al., 2022), optical stimulation techniques have an extremely high value in the neuromodulation field due to their high spatial accuracy and positional targeting. Among the optical stimulation techniques, the ability of infrared neural stimulation (INS) to activate or inhibit nerve cells without any genetic or chemical tissue modification provides better safety and clinical feasibility when compared with the other types of optical techniques (Rajguru et al., 2011). This form of neuromodulation has potential applications in diagnosing and treating many neurological and psychiatric disorders, such as dementia (Iaccarino et al., 2016), Parkinson's disease (Darlot et al., 2016), and depression (Tanaka et al., 2011). Nevertheless, the rational design and optimization of INS are hampered by the limited understanding of its neural effects.

Understanding how infrared (IR) stimulation affects neuronal activity and the mechanisms of interaction between the influences generated by IR light and neural tissue is necessary to address the question regarding the interaction between INS and neurons. A growing body of *in vitro* and *in vivo* evidence strongly suggests that laser is mediated by absorption of the local aqueous medium surrounding the heated cell to produce a thermal transient (Liu et al., 2009). INS regulates neuronal membrane capacitance and ion channel conductivity through the temperature-dependent mechanism generated by this thermal transient effect (Shapiro et al., 2012; Singh et al., 2019) and then modulates neuronal excitability. It is important to investigate the effects of physical field modulation on the neural network by considering the complexities of neuron types and their connections. However, only a few studies on the efficiency of INS at the neural network level were reported (Xia and Nyberg, 2019). Hence, the mechanism of interaction between the thermal effects produced by IR neural stimulation and the activity of neuronal populations is still not clearly elucidated, especially how the changes at one cell level can affect the network. Indeed, the network effect of neuromodulation has been shown to exist in other physical stimulations through experiments and computational models (Miyawaki et al., 2012; Di Lazzaro et al., 2018).

Computational modeling is a powerful tool for investigating the mechanisms of INS and for helping bridge research scales from a single cell to the network. A wealth of theoretical and numerical models on the interaction of INS with neural tissue exists. Most of them used spiral ganglion neurons (SGNs) as potential stimulation targets to explore the effects of IR stimulation on the level of isolated individual neurons. For example, acute *in vivo* experiments using gerbils to record optically evoked compound action potentials in the cochlea demonstrated that the auditory nerve could be stimulated by optical radiation (Littlefield et al., 2010). Some researchers accurately simulated neuronal responses by building a modified Hodgkin-Huxley (HH)-type model to predict the action potential threshold generated by SGN stimulation (Brown et al., 2021). Optical stimulation techniques can significantly improve cochlear implants hampered by a lack of spatial selectivity (Richardson et al., 2020). The above results showed that most studies were performed at the level of individual neuronal cells and did not address the dynamic activity of neuronal networks exposed to IR light. Therefore, considering the specific effects of photothermal effects on the complex neuronal network and illustrating the interaction between the photothermal effect and the neuronal network through the simulation results of the computational model are necessary.

In this study, we pursued a mixed strategy and developed a cortical neuronal network model by lumping both microscopic and macroscopic aspects to quantify the process of neuronal network response to IR light stimulation. The model combined excitatory and inhibitory neurons and synaptic structures, all of which were essential to accurately model the effects of IR neural stimulation. The present study aimed to investigate whether laser irradiation could regulate network activity. With this more complete model, we illustrated that the thermal effect in optical modulation affected the activity in individual neurons, as well as neuronal networks in a biphasic dose-response manner, thus providing a reasonable reference for biological experiments.

## Materials and methods

Based on neurophysiological features and experimental observations, the typical neuronal network model includes excitatory and inhibitory neurons, which are connected by excitatory and inhibitory synapses, respectively, forming a feedback circuit (Ocker et al., 2015). We focused on two levels, ion channels and membrane capacitance, and extended to the network structure to investigate the process of IR regulation on neurons. The model construction and its dynamics analysis from individual neurons to the neuronal network were as follows.

### Neuron model

As the basic element of a neuronal network, the neuron plays a fundamental role in modeling. Neuron modeling is the primary step in developing neuronal networks. The well-known Hodgkin-Huxley (HH) model was used in our neural network (Hodgkin and Huxley, 1952). The corresponding dynamic equation is as follows:


(1) $$\begin{array}{l} C_{m}\frac{dv_{m}}{dt}=-{\bar{g}}_{leak}(v_{m}-E_{leak})\\ -{\bar{g}}_{Na}m^{3}h(v_{m}-E_{Na})-{\bar{g}}_{K}n^{4}(v_{m}-E_{K})+I_{ext} \end{array}$$

where *C*_*m*_ is the membrane capacitance, *v*_*m*_ is the membrane potential, ${\bar{g}}_{leak}$, ${\bar{g}}_{Na}$, and ${\bar{g}}_{K}$ are maximal conductance of the leak, sodium, and potassium channels, respectively. *E*_*leak*_, *E*_*Na*_, and *E*_*K*_ are the reversal potentials, and *I*_*ext*_ is the external current injected into the membrane, i.e., background current.

The environment to which the neuronal cells are exposed generates temperature changes according to the rapid thermal transients generated by IR radiation in biological tissues. Therefore, a modified model of HH neurons was proposed through this temperature-dependent process. The improved model could visualize the kinetics process of neurons under the photothermal effect.

We extended the temperature influence factor ϕ(*T*) (Chandler and Meves, 1970), which affected neuronal activity by modulating the conductance and gating kinetics of ion channels. Thus, the firing process and dynamic changes of neurons under the premise of the thermal effect can be effectively simulated. Additionally, neuronal excitability is acutely affected by temperature through the changes in Nernst equilibrium potential (Kim and Connors, 2012). According to the original hypothesis of Hodgkin and Huxley, the activation *m*, *n* and inactivation *h* gating variables could be combined with the temperature coefficient ϕ(*T*), thus introducing temperature variables into the opening and closing rates of ion channels. Thus, the model of modulation of neuronal ion channels by photothermal effects is described by the following equations:


(2) $$\begin{array}{l} \alpha_{n}=\frac{0.032\phi \left(T\right)5}{\exp\left[\left(-48-v_{m}\right)/5\right]} \end{array}$$

(3) $$\begin{array}{l} \beta_{n}=0.5\phi \left(T\right)\exp\left[\left(-53-v_{m}\right)/40\right] \end{array}$$

(4) $$\begin{array}{l} \alpha_{m}=\frac{0.32\phi \left(T\right)4}{\exp\left[\left(-50-v_{m}\right)/4\right]} \end{array}$$

(5) $$\begin{array}{l} \beta_{m}=\frac{0.28\phi \left(T\right)5}{\exp\left[\left(-103-v_{m}\right)/5\right]} \end{array}$$

(6) $$\begin{array}{l} \beta_{h}=\frac{4\phi \left(T\right)}{1+\exp\left[\left(-23-v_{m}\right)/5\right]} \end{array}$$

(7) $$\begin{array}{l} \phi \left(T\right)=3^{\left(T-6.3\right)/10} \end{array}$$

where α_*n*_ and β_*n*_ are the opening and closing rates of the K^+^ channel, α_*m*_and β_*m*_ are the opening and closing rates for the activation gates of the Na^+^ channel, and α_*h*_ and β_*h*_ are the opening and closing rates for the inactivation gates of the Na^+^ channel, respectively.

The IR radiation not only thermally modulates the ion channel but also produces a temperature-dependent effect on the membrane capacitance. Early experimental studies demonstrated a correlation between capacitance (*C*_*m*_) and temperature (*T*) (Santos-Sacchi and Huang, 1998). Based on the ferroelectric Curie-Weiss law, the temperature-dependent effect of membrane capacitance could be represented visually and the temperature-capacitance relationship could be effectively fitted experimentally (Leuchtag, 1995). The fitting equation is described as follows:


(8) $$\begin{array}{l} C_{m}=C_{0}+\frac{k}{T_{c}-T} \end{array}$$

where *k* is capacitance constant; *C*_0_ is a constant membrane capacitance; and *T*_*c*_ is the Curie temperature of membrane capacitance. The Curie temperature of the membrane capacitor varied depending on the type of squid. Therefore, based on the data obtained from the HH model, the Curie temperature range was 31–50°C.

In addition, the photothermal effect also affects the size of the lipid bilayer, which in turn leads to changes in the membrane capacitance of the neurons. In conventional models, the capacitance would be assumed to be constant. However, recent studies demonstrated that under the condition of IR radiation, the change in capacitance caused a part of displacement current, with temperature dependence (Peterson and Tyler, 2012). As a result, we introduced the capacitive current component (Brown et al., 2021), which could be expressed as the time derivative of the membrane capacitance charge *C*_*m*_(*v*_*m*_−*V*_*s*_):


(9) $$\begin{array}{l} I_{m}=\left(v_{m}-V_{s}\right)\frac{dC_{m}}{dt} \end{array}$$

where *dC*_*m*_/*dt* denotes the laser-induced *dT*/*dt* as a function of the relational gradient *dC*_*m*_/*dT*. *V*_*s*_ is the asymmetric surface charge potential. The capacitive current component was well-fitted to the equation.

For the heat transfer effect of continuous wave laser, the increased temperature varied for different wavelengths, but a similar trend occurred in the case of the temperature change rate. As the irradiation time increased, the temperature gradient decreased significantly with respect to the initial value. Therefore, under the specified laser pulse conditions, *dC*_*m*_/*dt* was linearly proportional to *dT*/*dt* with a temperature-dependent capacitance factor *dT*/*dt* = 0.313%°C^−1^ (Plaksin et al., 2018). Thus, the capacitor current equation is read as follows:


(10) $$\begin{array}{l} I_{m}=3.13\times 10^{-3}\frac{dT}{dt}\left(v_{m}-V_{s}\right) \end{array}$$

**Figure 1:**
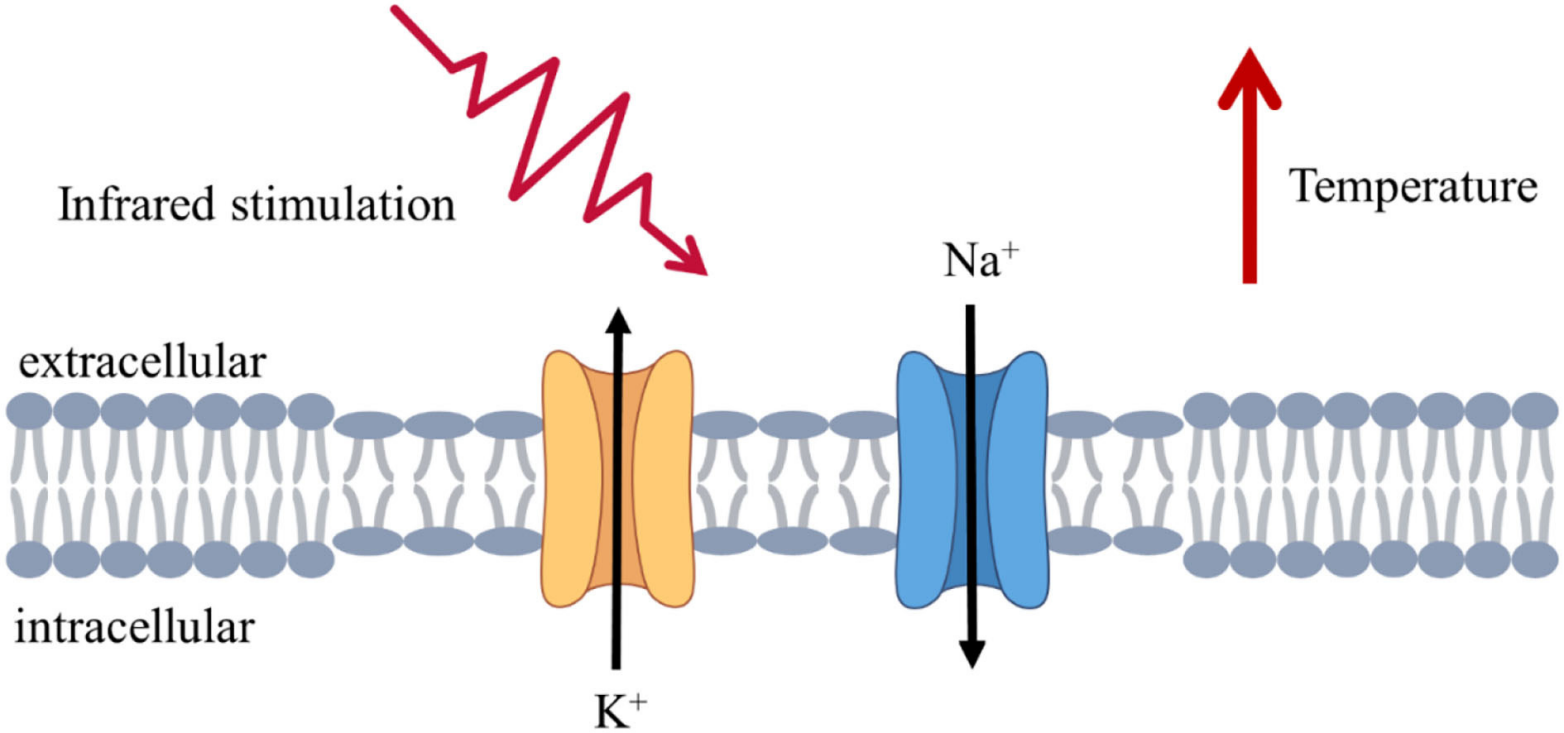
Schematic illustration of infrared stimulation at the cellular and molecular levels. Infrared irradiation caused changes in ion channel and membrane capacitance, increases in the rate of ion channel opening and closing, and changes in the thinning and larger area of the lipid bilayer, leading to changes in the response current.

The schematic illustration of IR regulation on ion channels and membrane capacitance is shown in Figure 1. The parameters (Hodgkin and Huxley, 1952) used in the neuronal model are listed in Table 1.

**Table 1:** Parameters used in the neuron model.

**Parameter**	**Description**	**Value**
*g*_*leak*_	Leak channel conductance	0.05 mS
*g*_*Na*_	Sodium channel conductance	50 mS
*g*_*K*_	Potassium channel conductance	30 mS
*E*_*Leak*_	Reversal potential of leakage channel	−60 mV
*E*_*Na*_	Reversal potential of Na^+^	90 mV
*E*_*K*_	Reversal potential of K^+^	−85 mV
*V*_*s*_	Asymmetric surface charge potential	28 mV
*V*_*th*_	Firing threshold	−63 mV
*V*_*rest*_	Resting potential	−70 mV
*I*_*ext*_	External current	1 pA
*k*	Capacitance constant	2.2
*C*_0_	Constant membrane capacitance	0.824 μF

### Synapse model

In neurophysiology, synapses are the sites where connections between neurons occur functionally and are also the key players in constituting models of complex neuronal networks. We used the synapse model originally proposed by Tsodyks and Markram to describe the dynamics of the synaptic terminal (Tsodyks et al., 1998; Barak and Tsodyks, 2007). The synaptic release process was achieved by the product of the variables *u*_*s*_ and *x*_*s*_, in which *u*_*s*_ represents the fraction of available neurotransmitter resources “docked” for release, and *x*_*s*_ is related to the proportion of total neurotransmitters that could be released. Upon the arrival of an action potential, *u*_*s*_ decayed to 0 at the 1/τ_*f*_ rate while *x*_*s*_ reinstated to 1 at the 1/τ_*r*_ rate. The process mimicked neurotransmitter depletion and reintegration and can be read by the following set of equations:


(11) $$\begin{array}{l} \frac{du_{s}}{dt}=\frac{-u_{s}}{\tau_{f}}+U_{0}\cdot \left(1-u_{s}\right)\cdot \delta \left(t-t_{K}\right) \end{array}$$

(12) $$\begin{array}{l} \frac{dx_{s}}{dt}=\frac{1-x_{s}}{\tau_{r}}-r_{s}\cdot \delta \left(t-t_{K}\right) \end{array}$$

where *U*_0_ is initial synaptic release probability at rest; released neurotransmitter resources from the presynaptic terminal can be calculated as follows:


(13) $$\begin{array}{l} r_{s}=u_{s}\cdot x_{s} \end{array}$$

Then, the neurotransmitter concentration *G*_*s*_ in the synaptic cleft is given by De Pitta and Brunel (2016):


(14) $$\begin{array}{l} \frac{dG_{s}}{dt}=-\Omega_{c}\cdot G_{s}+r_{s}\cdot Q_{c}\cdot Y_{T}\cdot \delta \left(t-t_{K}\right) \end{array}$$

where in neurotransmitter clearance rate, *Q*_*c*_ is vesicular vs. mixing volume ratio and *Y*_*T*_ is total vesicular neurotransmitter concentration. When a presynaptic action potential occurred, the postsynaptic neuron was responded by increasing corresponding excitability or inhibition conductance and then gave rise to postsynaptic currents. The fraction of postsynaptic receptors in the open state *r* can be described by the following first-order dynamic equation:


(15) $$\begin{array}{l} \frac{dr}{dt}=\alpha \cdot G_{s}\cdot \left(1-r\right)-\beta \cdot r \end{array}$$

where α and β are the forward and backward rate constants, respectively. Finally, α-amino-3-hydroxy-5-methyl-4-isoxazolepropionic acid (AMPA)- and N-methyl D-aspartate (NMDA)-mediated excitatory postsynaptic currents (EPSCs) are expressed by the following equations:


(16) $$\begin{array}{l} I_{AMPA}={\bar{g}}_{AMPA}\cdot r\left(t\right)\cdot \left(v_{m}-E_{AMPA}\right) \end{array}$$

(17) $$\begin{array}{l} I_{NMDA}={\bar{g}}_{NMDA}\cdot Mg\left(v_{m}\right)\cdot r\left(t\right)\cdot \left(v_{m}-E_{NMDA}\right) \end{array}$$

(18) $$\begin{array}{l} Mg\left(v_{m}\right)=\frac{1}{1+\exp\left(-0.062*v_{m}\right)\left[Mg^{2+}\right]/3.57} \end{array}$$

where $\bar{g}$ is the maximum synaptic conductance with ${\bar{g}}_{AMPA}$ = 0.35 nS, ${\bar{g}}_{NMDA}$ = 0.026 mS. *E* is the synaptic reversal potential with *E*_*AMPA*_ = *E*_*NMDA*_ = 0 mV. Noteworthily, NMDA receptor channels contain a voltage-dependent term representing magnesium (Mg^2+^) block with [Mg^2+^] = 1 mM (Jahr and Stevens, 1990). The parameters (De Pitta and Brunel, 2016) used in the synapse model are listed in Table 2.

**Table 2:** Parameters used in the synapse model.

**Parameter**	**Description**	**Value**
*U*_0_	Resting synaptic release probability	0.6
τ_*f*_	Facilitation time constant	0.3 s^−1^
τ_*r*_	Recovery time constant	0.5 s^−1^
*Y*_*T*_	Total vesicular neurotransmitter concentration	500 mM
Ω_*c*_	Neurotransmitter clearance rate	40 s^−1^
*Q*_*c*_	Vesicular vs. mixing volume ratio	0.005
α_*AMPA*_	AMPA forward rate constant	1.1 μM^−1^·s^−1^
β_*AMPA*_	AMPA backward rate constant	190 s^−1^
α_*NMDA*_	NMDA forward rate constant	0.072 μM^−1^·s^−1^
β_*NMDA*_	NMDA backward rate constant	6.6 s^−1^

### Neuronal network

The cerebral cortex is a multi-scale structure with local circuits interwoven to form a global network of remote connections. Within this complex network structure, neural activity propagates widely across temporal and spatial scales. The network model constructed in this study started from the microscale and took excitatory and inhibitory neurons as the basic components to respond to the photothermal effect of INS through synaptic interactions. Based on the neuroanatomical ratio of excitatory to inhibitory neurons (4:1) (Manos et al., 2021), the network model we designed comprised 3,200 excitatory neurons and 800 inhibitory neurons. The excitatory neurons with 5% of the connected weight enhanced signals, and the inhibitory neurons with 20% of the connected weight transmitted suppression signals.

**Figure 2:**
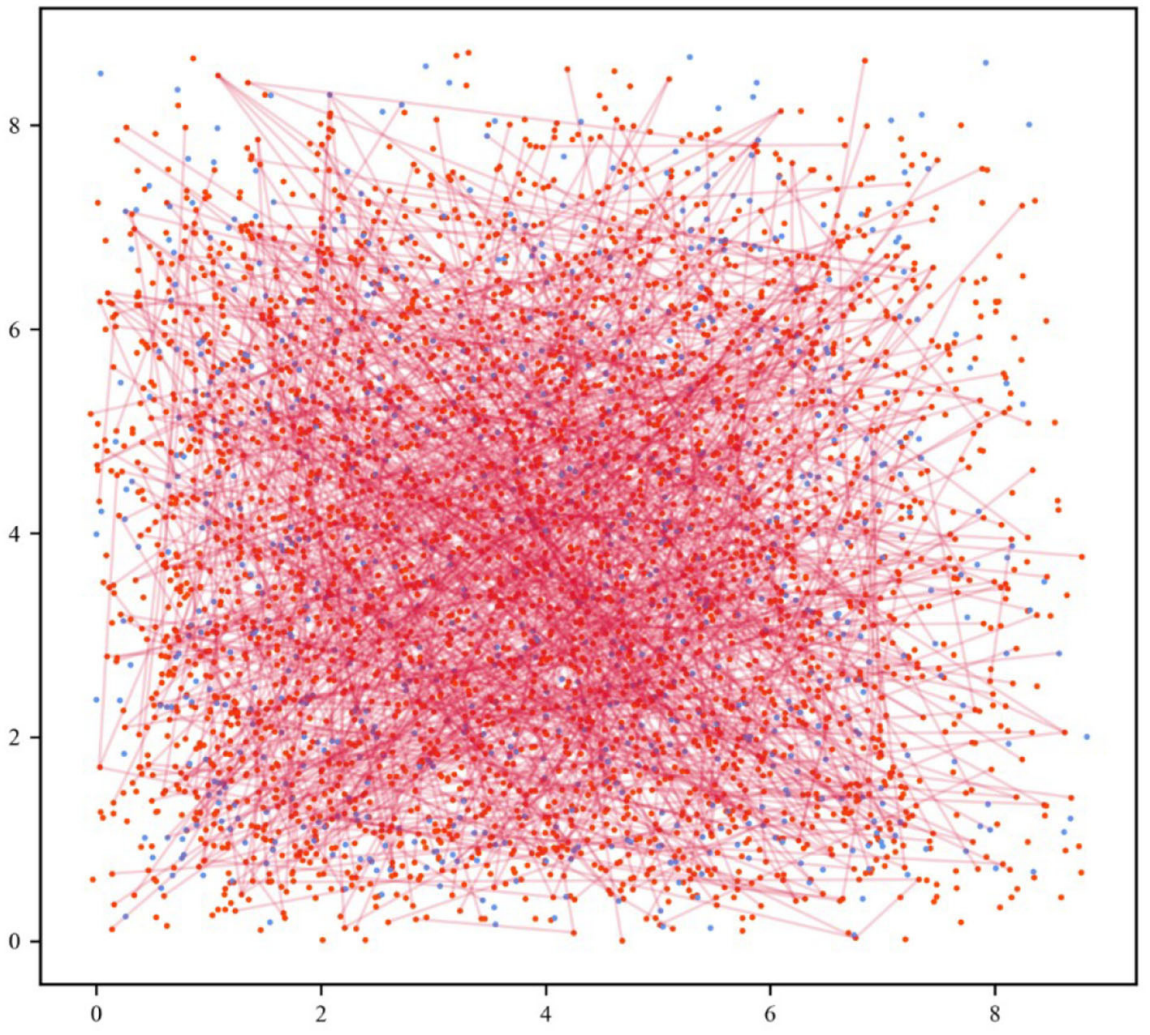
Neuronal network visualization in the Euclidean space. The 2D network diagram shows excitatory neurons in the red dots and inhibitory neurons in the blue dots. For the sake of clarity, the connection of 5% out of all excitatory synapses was selected at random (red lines).

Further, the cell populations were distributed in the Euclidean space to visualize and analyze the neuronal network. In the 2D map of the network, red represents excitatory neurons and blue represents inhibitory neurons, as shown in Figure 2. In cortical neuronal networks, excitatory inputs and inhibitory equivalents entered the cell together, allowing targeted transient or sustained opening of signal receptors. This tight coupling of excitatory and inhibitory signals exhibited a more intuitive state of network equilibrium (Jirsa, 2004). The model we developed was implemented in the Brian 2.0 simulator (Goodman and Brette, 2008; Stimberg et al., 2017).

## Results

### IR neural stimulation-induced temperature rise

The IR radiation is absorbed by the cellular tissue and converted into thermal energy (Wells et al., 2007a; Thompson et al., 2013). The temperature of the stimulation target increases, leading to temperature-dependent neuronal stimulation. The focus of this study was to investigate the spike activity and coding processes of neurons and neuronal networks in terms of the thermal effects generated by the action of IR light. Since the majority of the material in biological tissues is water, we first considered the process of temperature change produced by the irradiation of IR light in water. The data obtained from the experiments provided support for the simulation of neuronal networks.

**Figure 3:**
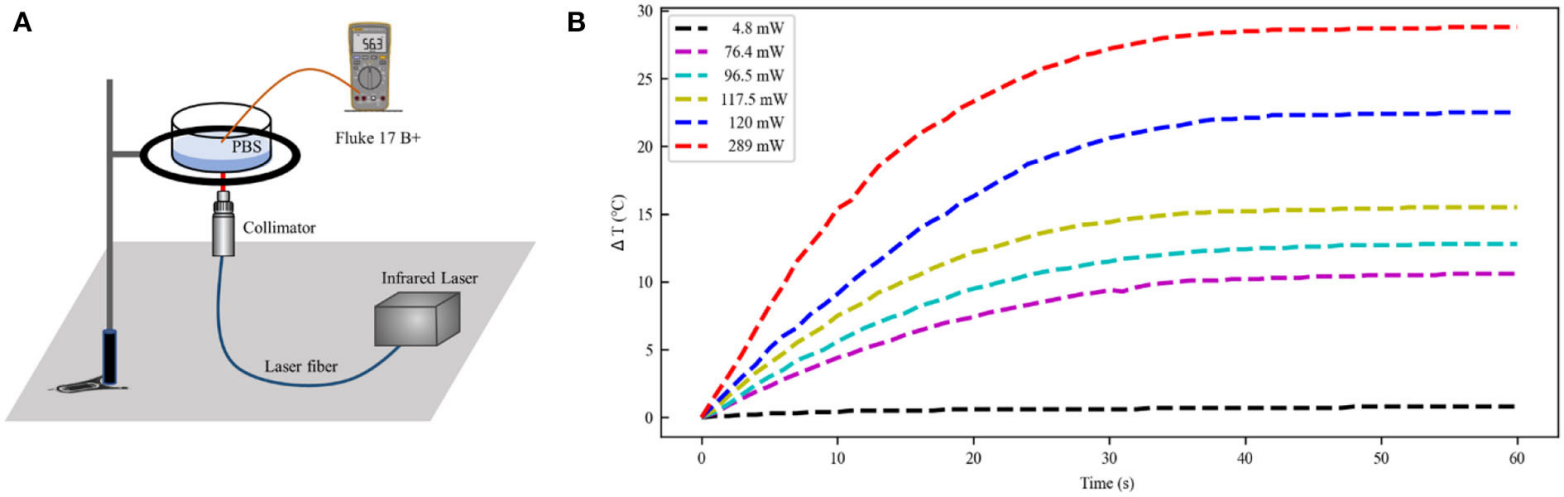
Temperature distribution at the different laser power. **(A)** The experimental setup of laser irradiation detection. **(B)** Temperature change curve over time caused by infrared light irradiation at 1,550 nm (initial temperature 21°C). PBS, phosphate buffered solution.

The schematic illustration of a laser irradiation detection device is shown in Figure 3A. Phosphate-buffered solution (PBS; Solarbio, China) of 0.5 ml was irradiated using IR laser 1,550 nm (Changchun New Industries Optoelectronics, China) in 24-well plates (Corning, USA) at room temperature. The optical stimulation was performed at the bottom of each well with a temperature probe (Fluke 17B+, USA) 10 mm away from the well. The power of the laser over the beam region was monitored by a Thorlabs (Thorlabs PM100D, USA). The temperature variation induced by laser irradiation is shown in Figure 3B. The increased temperature distribution ranged from 0.9 to 30°C at the different laser powers, in which the trends showed a rapid increase and gradual stabilization of temperatures (Xia and Nyberg, 2019). Consequently, the temperature increases in the INS computational model were primarily 10, 20, and 30°C.

### Spiking rhythms exposed to IR neural stimulation

The equations were inserted in editable format from the equation editor. We described the firing behavior of the neurons to verify whether IR-induced temperature changes could evoke neuron depolarization. A constant offset current with an amplitude of *I*_*ext*_ = 1 pA was injected into the neuronal model to induce tonic spikes in the neuronal action potential. We used neuronal spikes without photothermal effects as the original reference and the variation of the neuronal membrane potential in the experimentally probed temperature range was characterized.

**Figure 4:**
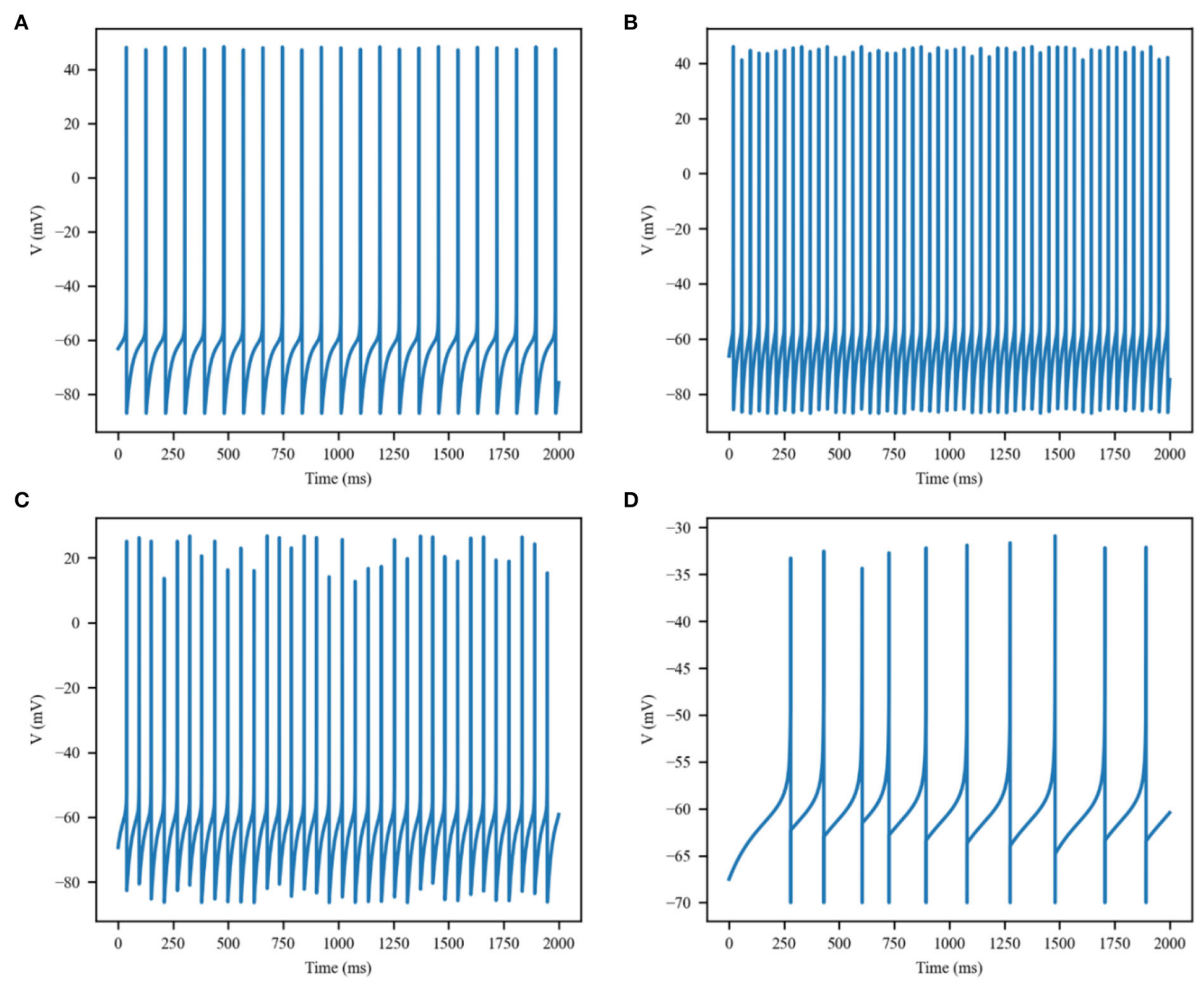
Membrane potential of single excitatory neurons under the action of increase in temperature by **(A)** 0, **(B)** 10, **(C)** 20, and **(D)** 30°C.

**Figure 5:**
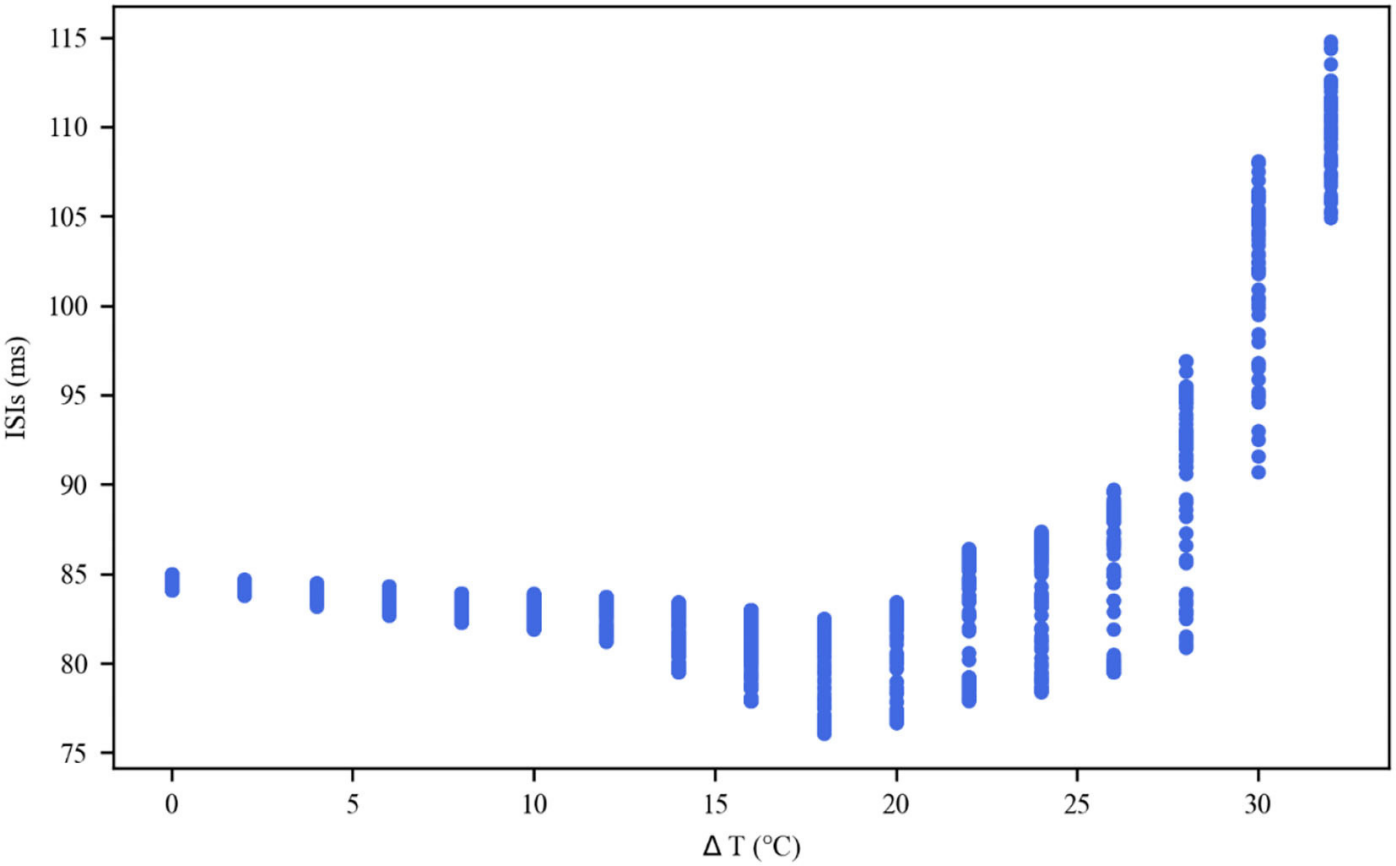
Inter-spike interval (ISI) sequences of neuronal spiking trains exposed to different temperatures.

**Figure 6:**
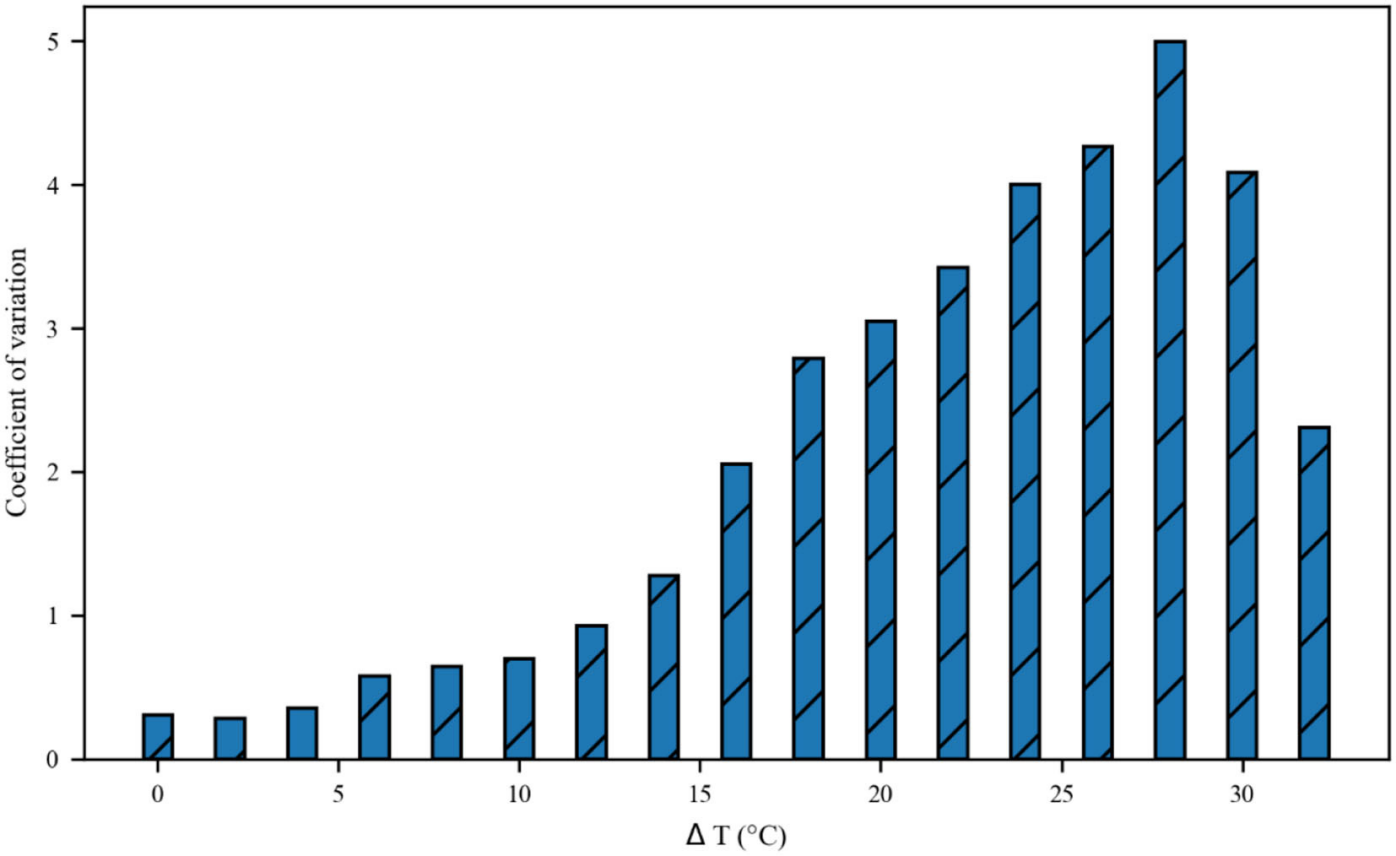
The Coefficient of Variance (CV) value of neurons inter-spike intervals exposed to different temperatures.

The thermal effect produced by IR light is interfered with the spike timing of neurons, as shown in Figure 4. The numbers of neuronal spikes from the recording time were 22, 52, 34, and 9 with the temperature increase of 0, 10, 20, and 30°C, respectively. Compared with no change in temperature, the neuronal spikes at 10 and 20°C were increased by 136.4 and 45.4%, respectively, and the spike count at 30°C was decreased by 59.1%. These results revealed an optical dose-dependent biphasic cell response. Figure 5 shows the inter-spike intervals (ISIs) changes in neuronal spiking trains evoked by different temperature conditions. ISIs were equally distributed with approximately the same value (84.5 ms) in the absence of optical stimulation. The variations in ISIs were related to the changes in neuronal spike time. With an increase in temperature (0°C < ΔT <20°C), the lower ISIs values represented a high neuronal spike count and the data presented irregularity. With increasing temperature (20°C < ΔT <30°C), the higher value of ISIs referred to sparse firing of neurons, indicating that the neuronal activity was inhibited, which was in tune with the results shown in Figure 4D. The large range of ISIs showed irregular neuron firing. Furthermore, we calculated and analyzed the Coefficient of Variation (CV) of neurons under different temperature changes, as shown in Figure 6. The results visually displayed the increasing trend of CV value with the increased temperature. In this process, the irregularity of interspike time became larger, and the neuronal activity became more active. When the temperature continued to rise, the CV value began to decrease, and the neuron activity decreased. Overall, the result of CV is consistent with the change of spike rate and shape of the single neuron during treatment with increased temperature. Except that the time course of an action potential characterized by ISIs and CV was affected, the result also shows the decrease of amplitudes of action potentials changing with temperature increase (Hodgkin and Katz, 1949).

Overall, these results indicated that the temperature changes of different intensities powerfully influenced neuronal spiking rhythms by the capacitive current and voltage-gated ion channels, and this effect was increased with increasing stimulus intensity.

### Modulation of presynaptic release and postsynaptic currents with IR neural stimulation

**Figure 7:**
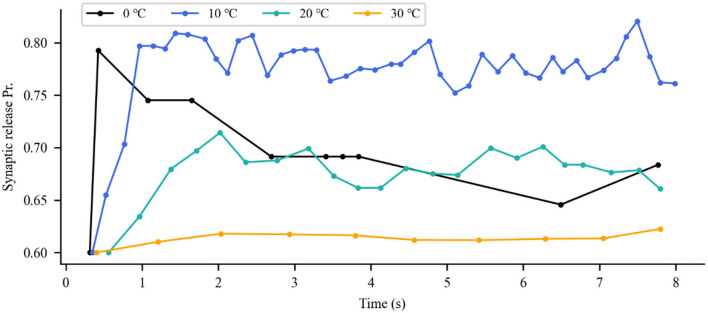
Variations in presynaptic glutamate release probability (Pr) in response to different temperatures. The dots represent each presynaptic release event.

**Figure 8:**
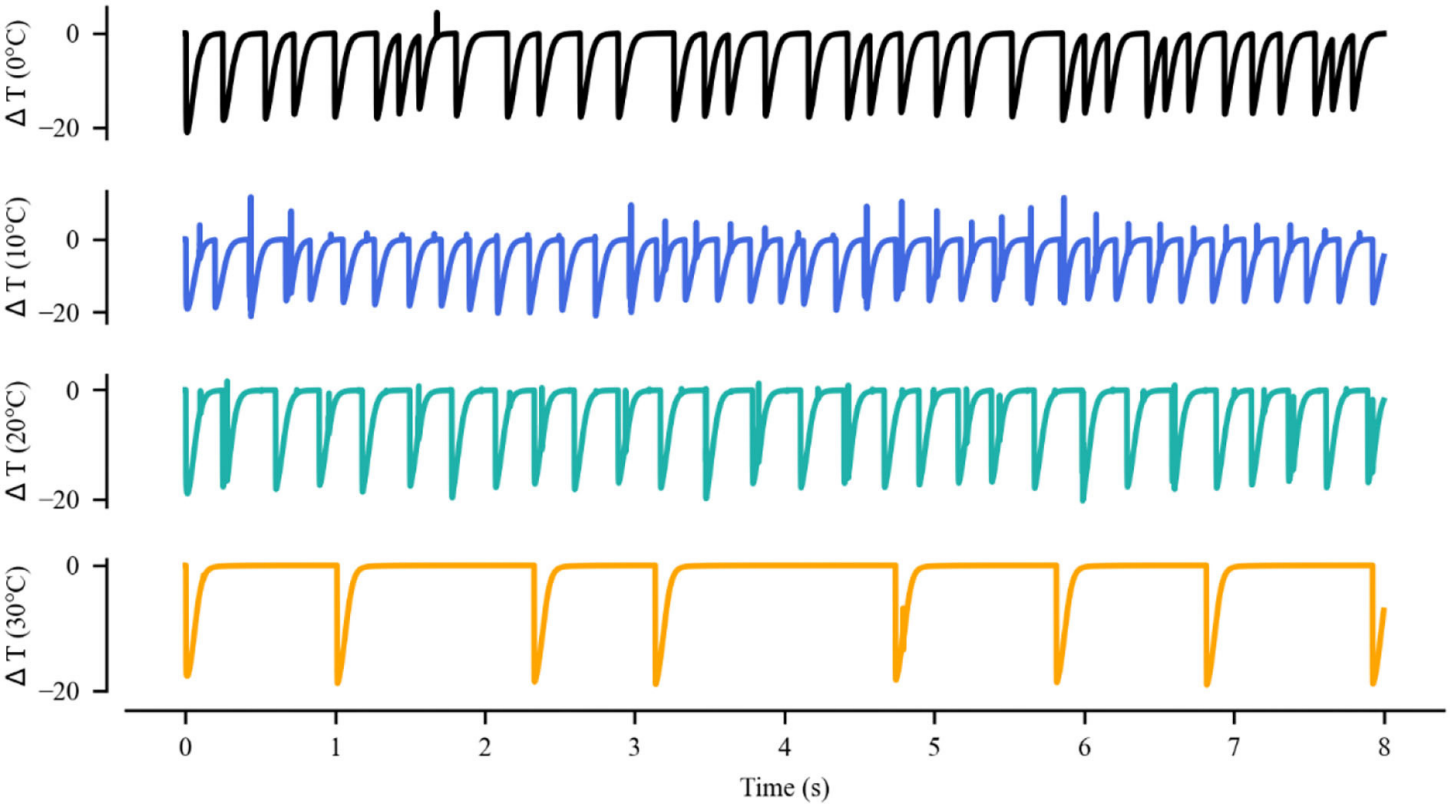
Variations in excitatory postsynaptic currents (EPSCs) evoked by glutamate released from the presynaptic terminal during treatment with different temperatures.

The aforementioned analysis revealed the impact of IR neural stimulation on neuronal activity. The neurotransmitters released from presynaptic terminals were changed with neuronal spike activity. Thus, we investigated the synaptic transmission response to the photothermal effects of IR neural stimulation. As previously reported in the literature, IR stimulation has the potential ability to modulate glutamate release by stimulating glutamatergic nerve endings (Amaroli et al., 2018). This scenario is illustrated in Figure 7, depicting the response of the synaptic model to a series of action potential changes induced by the photothermal influence. Increased temperature (10 and 20°C) robustly enhanced excitatory presynaptic release probability (Figure 7, blue and green lines). A slight temperature increase generated by IR laser light could stimulate vesicular neurotransmitter release. In the presence of a higher temperature at 30°C, the scenario was reversed (Figure 7, orange line), that is, photoinduced hyperthermia inhibited excitatory presynaptic release. Figure 8 shows that different temperatures affect the EPSCs under photothermal action with a running time of 10 s. At different temperatures, the discrepancy in postsynaptic activity was observed, which was in line with the dose of photothermal effect and the quantal size variability in presynaptic neurotransmitter release (Figure 7). The increased temperature induced by INS affected synaptic activity to a large extent when compared with the absence of photothermal stimulation. It could increase or decrease the frequency of neuronal spikes and affect synaptic efficacy and neural information processing.

### Impacts of IR neural stimulation exposure on a neuronal network

The connectivity of excitatory and inhibitory neurons as the basic ingredients is specified by synapses, which ultimately make up the interaction and co-regulation of a complex network structure. Akin to simulate the network structure in cortical neurons, the network is capable of displaying complex dynamics analysis behaviors.

**Figure 9:**
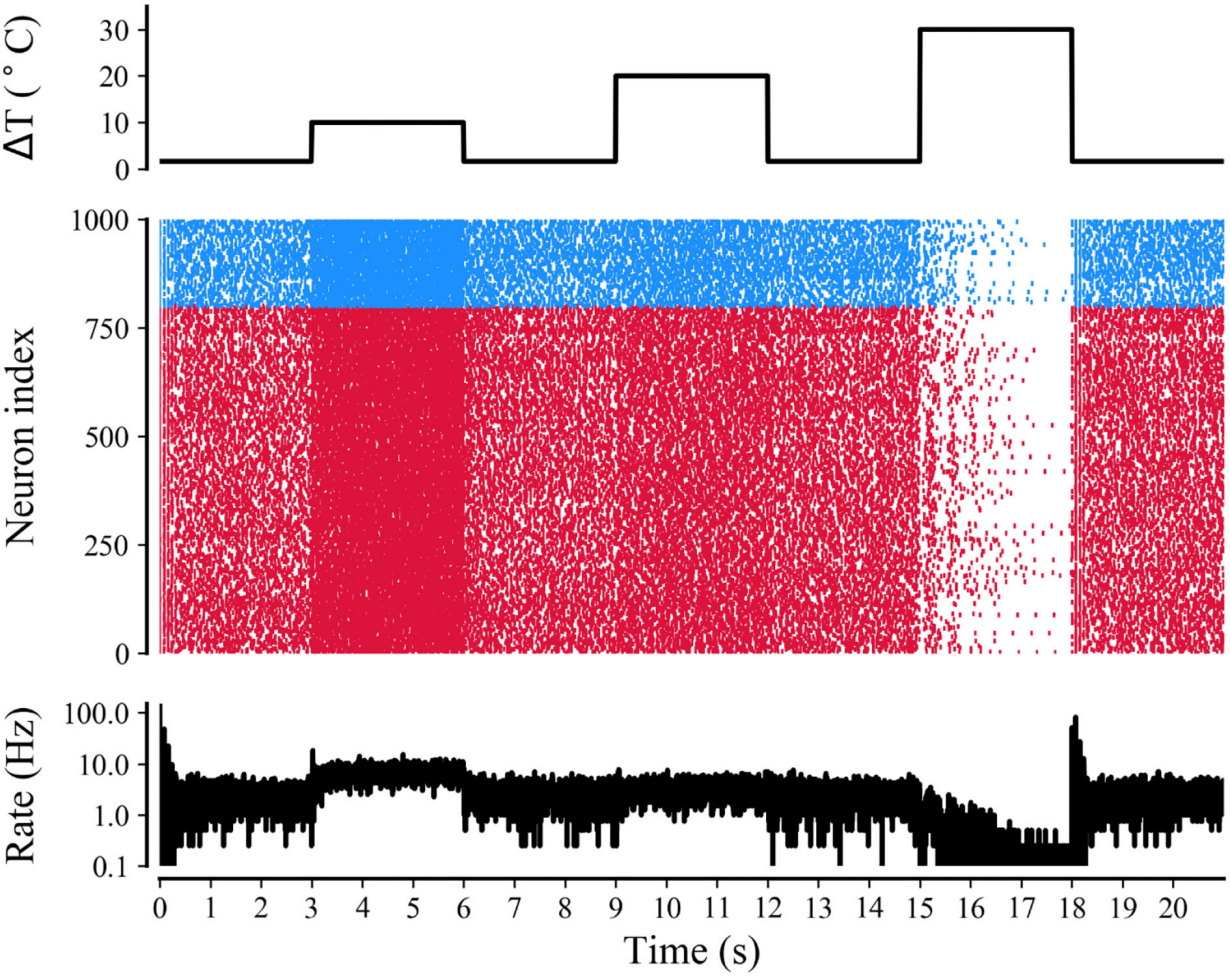
Raster plot and mean firing rate of neural activity during treatment with different temperatures. Simulations of neuronal network for a rectangular-pulse increase in temperature by 10, 20, and 30°C (top panel). The raster plot (middle panel) shows the spike activity of 25% of all excitatory (red) and inhibitory neurons (blue) of the network. The mean firing rate of the network is shown in the bottom panel.

**Figure 10:**
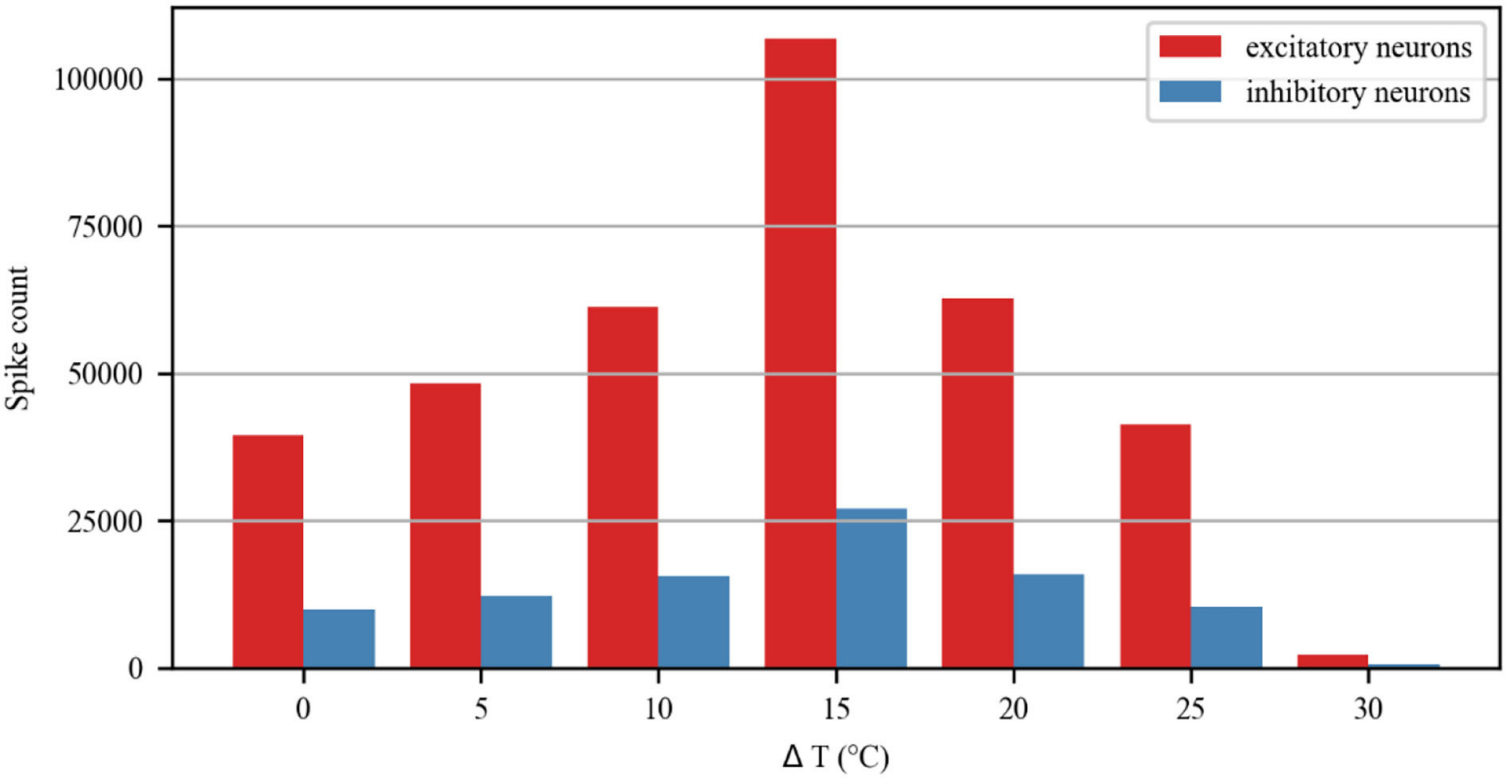
Spike count of excitatory and inhibitory neurons in the neuronal network during treatment with different temperatures.

The simulation of the neuronal network in Figure 9 shows a raster plot of the firing activity of 25% of the excitatory neurons (red) and inhibitory neurons (blue) in the network and in response to temporarily increasing external stimuli with 10, 20, and 30°C (rectangular stimulus change in the top panel). Prior to stimulus onset (t <3 s, increased 0°C), the neuronal ambient temperature was in a moderate situation. Therefore, the model was in a state of network equilibrium that included a network-averaged firing rate (bottom panel). For 3 < t <6 s and 9 < t <12 s (increasing to 10 and 20°C, respectively), all neurons were affected with increased temperature. The neuronal activity was significantly enhanced, as reflected by a denser raster plot and high-frequency population activity during this period. The external stimulus returned to its original value (at t = 6 and 12 s), and the neuronal firing returned to normal accordingly. With a temperature rise to 30°C (15 < t <18 s), the presented network raster plot and consequently the dynamic characteristics of the total firing rate were observed to show low-frequency population activity. The increased temperature and excitatory and inhibitory spike counts are shown in Figure 10. Thus, it was inferred that small temperature increases enhanced neuronal network activity, whereas higher temperatures inhibited the neuronal spike activity of the neuronal network. These results matched well with previous experimental observations (Xia and Nyberg, 2019), that is, beneficial at a low dose and harmful at a high dose.

## Discussion and conclusion

The potential utility of IR stimulation has been demonstrated by numerous experimental research studies. However, the lack of understanding of its underlying mechanisms has hindered its scientific and clinical applications. Based on neurophysiological findings, we designed a biophysical neuronal network model to mimic the interaction between the photothermal effect of IR neural stimulation and neurons. The simulation results of our study provided new insights to explore the response of neurons to optical stimulation.

Optical modulation can stimulate discrete groups of nerve fibers in a contact-free, damage-free, and artifact-free way. Regardless of the application, the interaction between the laser and the biological tissue results in light distribution and absorption, leading to photobiological effects. The generation of the photothermal effect is related to the transient irradiation process by IR light, which is a temperature dependent and transient mechanism. Our experiments are consistent with this conclusion by measuring the temperature increase of the PBS caused by the laser. After laser irradiation, the temperature increases exponentially, which reflects not only the temperature change but also the continuous transformation of temperature change rate with laser irradiation time. Combined with research and experiments (Ebtehaj et al., 2018; Ganguly et al., 2019), the thermo-induced capacitive current and the modulation of thermal-sensitive ion channels were modified in response to IR stimulation. The results suggested that IR light-induced thermal effects could regulate neuronal spikes, displaying the characteristics of optical dose dependence, that is, low-level laser enhances neuronal activity and high-level laser inhibits neuronal activity. These findings were akin to the expected optical dose-dependent biphasic cell response (Huang et al., 2009). Though the excitability of neurons depends on synaptic connections, our study here focused on individual neurons, suggesting that the laser can activate or inhibit the activity of neurons even without synaptic interactions or neuronal network properties. The result proves that the INS mechanism is mediated by temperature transients induced by IR absorption, and neural activation with laser light results from the thermal transient. In fact, mounting evidence indicates that many types of mild stresses, such as hyperthermia, hypothermia, and an altered pH, can directly or indirectly interfere with protein functions and signaling pathways in cells and then affect cell activity (Chen and Chiao, 2020). It is worth noting that the temperature change caused by the photothermal effect not only has an effect on voltage gating but also affects the reversal potential of sodium and potassium current based on the application of the Nernst equation (Yu et al., 2012).

A further interesting prediction of this model was the modulation of synaptic and network activity by IR neural stimulation. The results of the present study showed that moderately increased temperatures indeed enhanced neurotransmitter release probability and neuronal network activity in an optical dose-dependent manner. Irradiation-induced photothermal effect somehow stimulated the release of neurotransmitters at the synaptic level, thus facilitating the transmission of neural information. The efficiency of exocytosis is higher at a slightly increased temperature than without stimulation, while laser above a certain energy threshold reduced synaptic activity. This was in agreement with existing literature and experimental observations that nerve endings were sensitive to light, and the IR light was shown to induce amino acid neurotransmitters to release by stimulating glutamatergic or GABAergic nerve endings (Nouvian, 2007; Wells et al., 2007b; Ahmed et al., 2008; Feng et al., 2010; Amaroli et al., 2018), leading to the transmission or inhibition of nerve excitation. However, the specific neuron types and corresponding stimulus parameters have not been integrated into a unified framework. The reasons for this variability may be the different neuronal types in the brain, such as excitatory neurons and inhibitory neurons, or their subtypes vary in response to INS (Ahmed et al., 2008; Feng et al., 2010). Though precise stimulus parameters have not been validated, and the mechanism by which optical energy causes changes in synaptic function remains unclear, our simulation result showed that synapses could act as filters through a release-decreasing or release-increasing response to the external stimulus (Abbott and Regehr, 2004). The changes in synaptic structure and function often represent plasticity, which is the candidate mechanism for the change in brain function. Numerous studies on transcranial magnetic stimulation and transcranial electrical stimulation demonstrated that these two types of stimulations had effects on synaptic plasticity (Fritsch et al., 2010; Tang et al., 2017). However, optical stimulation working through direct or indirect effects on synapsis requires further exploration to elucidate the exact mechanism. In addition to the cell level, the photothermal effect of optical stimulation could regulate neural network firing rhythms in the manner of dose-dependent biphasic cell response. It is well-known that brain activity depends largely on collective phenomena, which arise from the complex networks connected through synapses. The network model structure can be adapted and adjusted by sensing external stimuli, which is a scale between the macroscopic brain and the microscopic neuron (Sporns et al., 2005). Numerical simulation results of our model show that the characteristics of changes in the network match well with the response of individual neurons and synaptic activity to temperature increase. The most important was that these scenarios were reversible, not permanent. Apart from photothermal effects, photochemical and optoacoustic effects or some other effects could also potentially contribute the neuronal response to IR neural stimulation (Kramer et al., 2009; Shi et al., 2022). Further experiments are needed to explore these possibilities.

In conclusion, this study developed a computational model for simulating the response of cortical neurons to IR neural stimulation and enabled the quantification of the effects of photothermal effects on individual neurons, synapses, and networks. The numerical simulation results demonstrated the importance of the photothermal effects of INS. This model will be optimized and integrated into a multi-scale model in the future to guide non-invasive brain stimulation programs.

## Data availability statement

The original contributions presented in the study are included in the article/supplementary material, further inquiries can be directed to the corresponding author/s.

## Author contributions

JW and LL contributed to the conception of the study and wrote an initial draft of the manuscript. HS and ZD organized the database and prepared the figures. JY, MZ, and XL reviewed and edited the manuscript and performed the statistical analysis. All authors contributed to manuscript revision, read, and approved the submitted version.

## Funding

Financial support for this work was provided by the National Natural Science Foundation of China (62006067), the Regional Innovation and Development Joint Fund of National Natural Science Foundation of China (U20A20224), and the Natural Science Foundation of Hebei Province (F2021201008) is gratefully acknowledged.

## Conflict of interest

The authors declare that the research was conducted in the absence of any commercial or financial relationships that could be construed as a potential conflict of interest.

## Publisher's note

All claims expressed in this article are solely those of the authors and do not necessarily represent those of their affiliated organizations, or those of the publisher, the editors and the reviewers. Any product that may be evaluated in this article, or claim that may be made by its manufacturer, is not guaranteed or endorsed by the publisher.
